# Cold Extremities and Underweight as a Combined Risk Profile for Open-Angle Glaucoma: A Population-Based Cross-Sectional Study in Korea

**DOI:** 10.3390/medicina62071411

**Published:** 2026-07-21

**Authors:** Kwang-Ho Bae, Youngseop Lee, Man Young Park, Ho-Yeon Go, Ilkoo Ahn, Yong-Sik Park

**Affiliations:** 1 KM Data Division, Korea Institute of Oriental Medicine, Daejeon 34054, Republic of Korea; 2 Digital Health Research Division, Korea Institute of Oriental Medicine, Daejeon 34054, Republic of Korea; 3 National Institute for Korean Medicine Development, Gyeongsan 38540, Republic of Korea; 4 Yonsei Best Ophthalmic Clinic, Jongno-gu, Seoul 03104, Republic of Korea

**Keywords:** open-angle glaucoma, Flammer syndrome, cold extremities, underweight, population-based study, predictive screening, hypersensitivity

## Abstract

*Background/Objectives*: Glaucoma is one of the leading causes of irreversible blindness worldwide. Open-angle glaucoma (OAG) predominates in East Asian populations, with normal-tension glaucoma accounting for the majority of cases. Although intraocular pressure remains the most established risk factor, non-pressure-dependent mechanisms, including ocular vascular dysregulation, warrant further investigation. Cold extremities, a hallmark of Flammer syndrome reflecting primary vascular dysregulation, and underweight (body mass index [BMI] < 18.5 kg/m^2^) are each independently associated with OAG; however, their combined effect has not been examined at the population level. *Materials and Methods*: Using nationally representative data from the Korea National Health and Nutrition Examination Survey 2008–2012 (*n* = 8135; controls 7418, OAG 717), participants aged ≥ 50 years with measured intraocular pressure within the normal range (5–21 mmHg) at the time of examination were classified into four groups: non-underweight without cold extremities (N-N, reference), cold extremities only (CEO), underweight only (UWO), and underweight with cold extremities (UW+CE). Complex sample logistic regression was adjusted for sex, age, diabetes mellitus, mean arterial pressure, intraocular pressure, and spherical equivalent. *Results*: UW+CE was associated with significantly higher odds of OAG than N-N (adjusted odds ratio [OR] = 2.10, 95% confidence interval: 1.14–3.88, *p* = 0.018), whereas neither CEO (OR = 1.10, *p* = 0.431) nor UWO (OR = 1.54, *p* = 0.170) was significantly associated individually. This association with UW+CE remained robust across alternative BMI thresholds and after further adjustment for socioeconomic, lifestyle, and cardiometabolic variables. *Conclusions*: Co-occurring cold extremities and underweight status were associated with a combined hemodynamic risk profile for OAG, suggesting their potential utility as clinically accessible noninvasive markers for the early identification of highly vulnerable individuals and informing proactive glaucoma screening strategies.

## 1. Introduction

Glaucoma is one of the leading causes of irreversible blindness worldwide, with approximately 64.3 million people aged 40–80 years affected as of 2013; this number is projected to reach approximately 111.8 million by 2040 as the global population ages [1]. This increase is expected to be disproportionately concentrated in Asia and Africa compared to other regions [1]. A nationally representative study conducted in Korea reported that the prevalence of primary open-angle glaucoma (POAG) among adults aged ≥ 40 years was 4.7% and exhibited a clear age-dependent increase, as follows: 6.2% in individuals in their 60s, 8.2% in those in their 70s, and 8.9% in those aged ≥ 80 years [2]. Despite this high prevalence, the awareness rate of diagnosis was only 8.0%, underscoring the need for early detection and systematic screening strategies targeting high-risk populations [2]. Open-angle glaucoma (OAG) is the most common subtype of glaucoma, and intraocular pressure (IOP) is its best-established risk factor. However, normal-tension glaucoma (NTG), which develops within the normal IOP range (≤21 mmHg), is the predominant subtype of OAG, particularly in East Asian populations [1,2,3], making the identification of non-IOP-dependent risk factors a clinically important challenge. Previously reported non-IOP-dependent risk factors include older age [1], myopia [4,5], diabetes mellitus (DM) [6], low ocular perfusion pressure [7], family history [7,8], and racial predisposition [1,9].

Among these non-IOP-dependent risk factors, underweight or low body mass index (BMI) has been consistently reported as an independent risk factor for OAG across diverse ethnic groups and study designs [2,10,11]. Cold extremities have also been reported as a factor associated with OAG, particularly NTG, because they are a clinical marker of vascular dysregulation [12] and have been linked to various chronic conditions [13,14]. Cold extremities are not uncommon in the Korean adult population, with a reported prevalence of 21.6% for cold hands and 23.0% for cold feet [15], making the condition a relevant public health-related concern and necessitating an investigation of the combined effect of underweight and cold extremities on OAG risk at the population level. These two factors can be conceptually integrated within the framework of Flammer syndrome (FS), a primary vascular dysregulation syndrome characterized by a core clinical phenotype comprising cold extremities, hypotension, and low BMI [16,17]. Moreover, FS may contribute to the pathogenesis of OAG through impaired ocular blood flow autoregulation. This conceptual framework suggests that underweight and cold extremities may be independently associated with OAG and that their co-occurrence may amplify hemodynamic vulnerability. However, previous studies have largely relied on hospital-based samples, and no studies have verified this association at the population level.

Therefore, we aimed to conduct the first population-level study to examine whether the combination of underweight and cold extremities is independently associated with the risk of OAG using data from the Korea National Health and Nutrition Examination Survey (KNHANES) cycles IV and V (2008–2012). KNHANES IV–V is the only nationally representative dataset that simultaneously incorporates data from the cold extremities questionnaire and ophthalmic examinations [18]. In contrast to previous studies that used the same dataset during specific periods and focused on individual risk factors [2,19,20,21,22], the present study establishes a novel analytical framework focused on the combined risk profile of underweight and cold extremities, restricts IOP to the normal range (5–21 mmHg), and applies strict pure-control criteria. The objective of this study was to provide evidence that may contribute to the early identification of individuals highly vulnerable to OAG using concurrent underweight and cold extremities as markers. Additionally, this evidence aims to inform the development of glaucoma screening strategies.

## 2. Materials and Methods

### 2.1. Study Design and Data Source

This population-based cross-sectional study was conducted between 2008 and 2012 using data from the KNHANES IV (2008–2009) and V (2010–2012) cycles. The KNHANES is a nationally approved statistical survey overseen by the Korea Disease Control and Prevention Agency (KDCA) designed to assess the health and nutritional status of a representative sample of community-dwelling Korean nationals. The survey adopts a rolling sampling design involving stratified multistage probability cluster sampling, through which approximately 10,000 individuals are selected to participate annually, such that the results of each survey year are representative of the entire Korean population. Detailed information on the data structure and sampling design of the KNHANES has been provided elsewhere [23]. Ophthalmic examinations incorporating the cold extremities questionnaire were introduced during the second half of 2008 and were conducted throughout KNHANES IV–V (2008–2012). The methodological details of the ophthalmic examinations have been published separately [18]. The KNHANES was conducted in accordance with the ethical principles of the Declaration of Helsinki and received approval from the Institutional Review Board (IRB) of the KDCA (IRB nos. 2008-04EXP-01-C, 2009-01CON-03-2C, 2010-02CON-21-C, 2011-02CON-06-C, and 2012-01EXP-01-2C). The use and analysis of the KNHANES data in the present study were approved by the IRB of the Korea Institute of Oriental Medicine (IRB no. I-2409/009-001; approved 13 September 2024). The raw KNHANES data used in this study are publicly available and provided free of charge upon researcher application at https://knhanes.kdca.go.kr/ (accessed on 4 June 2026). This study was reported in accordance with the Strengthening the Reporting of Observational Studies in Epidemiology (STROBE) guidelines for cross-sectional studies.

### 2.2. Study Population

Given the markedly increased prevalence of OAG among individuals aged ≥ 50 years [1,2], a base population of 17,117 individuals from this age group was extracted from the total of 45,811 KNHANES IV–V (2008–2012) participants. Subsequently, the final analytical sample was obtained through sequential exclusion using the following steps.

First, participants with missing data were excluded, including those with missing responses to the cold extremities questionnaire, anthropometric measurements (BMI, blood pressure), diabetes-related indices, spherical equivalent (SE), IOP, anterior chamber angle assessment, optic disc findings (cup-to-disc ratio [CDR], retinal nerve fiber layer [RNFL] defect, disc hemorrhage, inferior-superior-nasal-temporal [ISNT] rule), or visual field test results.

Second, in order to restrict the analytical focus to the normal IOP range, participants were excluded based on the following ophthalmic criteria: suspected angle closure (peripheral anterior chamber depth ≤ one-quarter of corneal thickness) and IOP ≤ 5 mmHg (ocular hypotony) or >21 mmHg (elevated IOP) [3,24].

Third, for diagnostic clarity, participants with suspicious optic disc findings who did not fulfill the International Society of Geographical and Epidemiological Ophthalmology (ISGEO) Category I or II diagnostic criteria for glaucoma were excluded from both the glaucoma and control groups to minimize misclassification bias.

**Figure 1:**
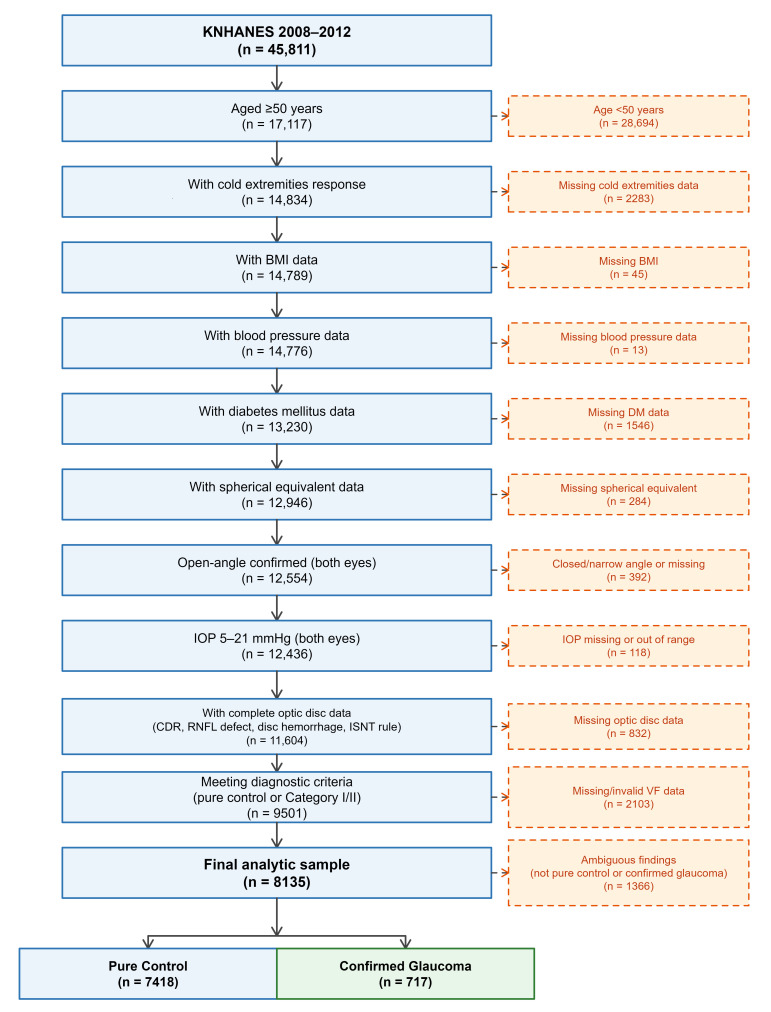
Flowchart of participant selection from the Korea National Health and Nutrition Examination Survey (KNHANES) 2008–2012 dataset. Ambiguous findings refer to suspicious optic disc findings in participants who did not fulfill the International Society of Geographical and Epidemiological Ophthalmology Category I or II diagnostic criteria for glaucoma. To minimize misclassification bias, these participants were excluded from both the glaucoma and control groups. Abbreviations: IOP, intraocular pressure; CDR, cup-to-disc ratio; RNFL, retinal nerve fiber layer; ISNT, inferior-superior-nasal-temporal; VF, visual field.

The final analytical sample comprised 8135 participants (controls: 7418; glaucoma: 717) (Figure 1). The excluded participants were older and had a lower BMI than those who were included. However, there was no significant difference in the prevalence of cold extremities or in the distribution of groups combining BMI and cold extremities between the two groups. A detailed comparison of the general and clinical characteristics of the included and excluded participants is provided in Supplementary Table S1.

### 2.3. Ophthalmic Examination and Glaucoma Diagnosis

Ophthalmic examinations were performed at mobile examination centers by ophthalmologists or ophthalmology residents trained by the Epidemiological Survey Committee of the Korean Ophthalmological Society. Detailed examination procedures followed the previously described methodology [18].

The specific examination components were as follows: Visual acuity and refraction were measured using an auto kerato-refractometer (KR-8800; Topcon, Tokyo, Japan), and SE was calculated as the sum of the spherical value and half the cylindrical value. IOP was measured once in each eye (right eye first, then left eye) using a Goldmann applanation tonometer (Haag-Streit AG, Koeniz, Switzerland); to minimize measurement error, the mean of both eyes was used in the present analysis. Anterior chamber angle assessment was performed using the Van Herick method with a slit lamp (BM 900; Haag-Streit AG, Koeniz, Switzerland) and an anterior chamber depth exceeding one-quarter of the corneal thickness, which was defined as an open angle. Fundus photography was performed using a non-mydriatic digital fundus camera (TRC-NW6S; Topcon, Tokyo, Japan) to capture 45-degree images centered on the macula.

Frequency doubling technology (FDT) perimetry (Humphrey Matrix; Carl Zeiss Meditec Inc., Dublin, CA, USA) was performed using the N-30-1 screening protocol and was administered selectively only when at least one of the following criteria was met: (1) IOP > 21 mmHg, (2) vertical or horizontal CDR ≥ 0.5, (3) optic disc hemorrhage, (4) RNFL defect, or (5) violation of the ISNT rule (inferior > superior > nasal > temporal). If the FDT results were unreliable (fixation error or false-positive error > 1), a repeat test was performed.

OAG classification was performed by a board-certified ophthalmologist using the ISGEO diagnostic criteria [25], according to the methodology reported in previous KNHANES-based studies [20,26]. Specifically, OAG was defined as the presence of an open angle confirmed by Van Herick slit-lamp examination (peripheral anterior chamber depth > one-quarter of the corneal thickness) in conjunction with the following criteria.

Category I (structural and functional evidence) was defined as follows: reliable FDT results (fixation error and false-positive error ≤ 1) indicating a functional abnormality (sensitivity loss at one or more locations) present along with at least one of the following structural criteria: (1) vertical or horizontal CDR ≥ 0.7 or vertical CDR asymmetry ≥ 0.2 (both at the 97.5th percentile of the KNHANES normal population); (2) optic disc hemorrhage; or (3) RNFL defect [20].

Category II (structural evidence only) was defined as follows: absent or unreliable FDT results (fixation error or false-positive error ≥ 2) along with at least one of the following structural criteria: (1) vertical CDR ≥ 0.9 or vertical CDR asymmetry ≥ 0.3 (both at the 99.5th percentile of the KNHANES normal population); (2) RNFL defect violating the ISNT rule [20].

Pure controls were defined as participants fulfilling all of the following criteria in both eyes: (1) IOP ≤ 21 mmHg; (2) open angle; (3) vertical and horizontal CDR < 0.5 and CDR asymmetry < 0.2 (participants with CDR ≥ 0.5 were classified as glaucoma suspects in the KNHANES and referred for FDT perimetry [26] and were therefore excluded from the pure control group); (4) absence of RNFL defect and optic disc hemorrhage; and (5) satisfaction of the ISNT rule.

### 2.4. Definitions of Key Variables

Cold extremities were evaluated using a self-reported questionnaire item from the KNHANES ophthalmic survey [18] that assessed both cold extremities and migraine simultaneously (translated from the original Korean): “Have you ever suffered from or are you currently experiencing cold extremities (cold sensations in your hands or feet) or migraines (throbbing headache on one side)?” The available responses were 1 (cold extremities), 2 (migraine), 3 (both), and 4 (neither). Participants who selected options 1 or 3 were classified as having cold extremities (present), whereas those who selected options 2 or 4 were classified as not having cold extremities (absent).

According to the World Health Organization (WHO) criteria, underweight was defined as a BMI < 18.5 kg/m^2^ [27]. BMI was calculated as body weight (kg) divided by height squared (m^2^) using standardized anthropometric measurements from the KNHANES health examination data.

The combined BMI–cold extremities groups were assigned as follows: participants were classified into four groups based on the presence or absence of underweight and cold extremities: N-N (non-underweight without cold extremities; reference group), CEO (cold extremities only: non-underweight with cold extremities), UWO (underweight only: underweight without cold extremities), and UW+CE (underweight with cold extremities).

DM was defined as a fasting blood glucose level ≥ 126 mg/dL, current use of oral hypoglycemic agents or insulin, or a diagnosis of diabetes made by a physician [28].

The mean arterial pressure (MAP) was calculated as the diastolic blood pressure + (systolic blood pressure − diastolic blood pressure)/3. Blood pressure was measured three times in the seated position using a standardized mercury sphygmomanometer, and the mean of the second and third measurements was used [19].

The mean ocular perfusion pressure (MOPP) was calculated as (2/3 × MAP) − IOP [7].

The mean IOP of both eyes was used [29].

Myopia was categorized based on the SE. A more negative SE value is indicative of a greater degree of myopia. In the analysis, an increasing SE in the myopic direction (larger absolute negative value) corresponded to an increasing risk. Myopia was categorized as follows: no myopia (SE ≥ −0.5D), low myopia (−0.5D > SE ≥ −3.0D), moderate myopia (−3.0D > SE ≥ −6.0D), and high myopia (SE < −6.0D) [20].

### 2.5. Statistical Analysis

As stratified multistage cluster sampling is used in the KNHANES, complex sample analysis incorporating the survey design was applied to all analyses [23]. Sample weights, strata, and clusters were included to obtain estimates representative of the non-institutionalized Korean population. Continuous variables are presented as mean ± standard error, and categorical variables are presented as weighted percentage (%) with standard error.

Between-group comparisons were performed using the complex sample general linear model for continuous variables and the Rao-Scott chi-square test for categorical variables. For the four-group comparisons, overall group differences were first tested; when a significant overall difference was identified for continuous variables, pairwise comparisons against the N-N group were performed using the sequential Bonferroni correction. For categorical variables, only overall significance was indicated, as pairwise post hoc comparisons are not directly supported for categorical variables in complex survey designs.

To assess the associations of cold extremities and underweight with OAG, complex sample binary logistic regression was performed. Three sequential models were constructed: a crude (unadjusted) model, Model 1 (adjusted for sex and age), and Model 2 (additionally adjusted for DM, MAP, IOP, and SE). Covariates in Model 2 were selected based on established risk factors for OAG [5,6,7,25] and the methodology used in previous KNHANES-based studies [2,19].

The additive interaction between cold extremities and underweight was assessed using the following three measures proposed by Knol and VanderWeele [30]: the relative excess risk due to interaction (RERI), attributable proportion due to interaction (AP), and synergy index (S-index). The 95% confidence intervals (CIs) for these measures were estimated using the delta method. RERI > 0, AP > 0, and S > 1 indicated a positive additive interaction. Multiplicative interaction was tested by adding an interaction term for cold extremities and underweight status to the logistic regression model.

To evaluate the influence of the BMI threshold used to define underweight, odds ratios (ORs) for the UW+CE group were estimated across BMI cutoff values ranging from <17.0 to <19.5 kg/m^2^ in increments of 0.5 kg/m^2^. All models included the same covariates as those in Model 2.

Sensitivity analyses included stratification by the ISGEO diagnostic category (Categories I and II separately) and the construction of a fully adjusted model that additionally incorporated alcohol consumption, smoking status, household income, education level, physical activity (metabolic equivalent of task, MET), hypercholesterolemia, hypertension, low high-density lipoprotein cholesterolemia, hypertriglyceridemia, and migraine. The full model was used to perform a supplementary analysis only because the increased number of covariates relative to the limited number of glaucoma events in the UW+CE group may violate the events-per-variable principle, and the missing data for the additional variables resulted in a reduced analytical sample. Consequently, Model 2, which included only established OAG risk factors, was used for the primary analysis. Multicollinearity among covariates was assessed using the variance inflation factor (VIF); all variables showed VIF < 2.0 (range: 1.017–1.047), confirming the absence of multicollinearity. All statistical analyses were performed using SPSS version 28.0 (IBM Corp., Armonk, NY, USA). Figures were generated using the ggplot2 package (version 4.0.2) in R (version 4.5.3; R Foundation for Statistical Computing, Vienna, Austria). Statistical significance was set at a two-sided *p* < 0.05.

## 3. Results

### 3.1. Participant Characteristics

After applying the inclusion and exclusion criteria to the 45,811 KNHANES 2008–2012 participants, a final analytical sample of 8135 individuals was obtained (Figure 1), comprising 7418 controls and 717 patients with glaucoma.

The glaucoma group was significantly older than the control group (63.96 vs. 60.89 years, *p* < 0.001) and included a larger proportion of men (54.2% vs. 44.3%, *p* < 0.001). Systolic blood pressure, diastolic blood pressure, MAP, IOP, and MOPP were all significantly higher in the glaucoma group (all *p* < 0.001), and SE was significantly lower in the glaucoma group, indicating a greater degree of myopia (−0.69 vs. −0.23D, *p* < 0.001). The prevalence of DM was also significantly higher in the glaucoma group (21.7% vs. 15.5%, *p* = 0.001). Although no significant difference in continuous BMI was observed between the two groups (23.87 vs. 24.16 kg/m^2^, *p* = 0.057), the proportion of underweight participants (BMI < 18.5 kg/m^2^) was significantly higher in the glaucoma group (4.5% vs. 2.3%, *p* = 0.001). The prevalence of cold extremities did not differ significantly between the two groups (24.0% vs. 23.2%, *p* = 0.685) (Table 1).

### 3.2. OAG Prevalence Across BMI–Cold Extremities Groups

The baseline characteristics of the participants in the four BMI–cold extremities groups are presented in Table 2. OAG prevalence differed significantly across the four groups (*p* = 0.016) as follows: 8.3% (unweighted *n* = 535) in the N-N group, 8.4% (unweighted *n* = 151) in the CEO group, 14.0% (unweighted *n* = 15) in the UWO group, and 17.7% (unweighted *n* = 16) in the UW+CE group. The CEO, UWO, and UW+CE groups were all significantly older than the N-N group (*p* = 0.039, *p* < 0.001, and *p* = 0.001, respectively), and the BMI was significantly lower in all three groups than in the N-N group (all *p* < 0.001). Diastolic blood pressure was significantly lower in the CEO (77.38 mmHg, *p* < 0.001), UWO (73.99 mmHg, *p* < 0.001), and UW+CE (74.94 mmHg, *p* < 0.001) groups than in the N-N group (78.87 mmHg), and MAP showed significant differences in the same direction (CEO, *p* < 0.001; UWO, *p* = 0.003; UW+CE, *p* = 0.035). MOPP was also significantly lower in all three groups than in the N-N group (49.08 mmHg) (CEO, *p* = 0.004; UWO, *p* = 0.016; UW+CE, *p* = 0.016). Although the overall group differences in systolic blood pressure and IOP were significant (*p* = 0.046 and *p* = 0.026, respectively), the results of pairwise comparisons with the N-N group did not reach significance. The sex distribution differed significantly across the four groups (*p* < 0.001), with a larger proportion of women in the CEO group (70.8%) and a larger proportion of men in the UWO group (71.3%). No significant differences in myopia grade or DM prevalence were observed between the groups.

### 3.3. Association Between BMI–Cold Extremities Status and OAG

**Figure 2:**
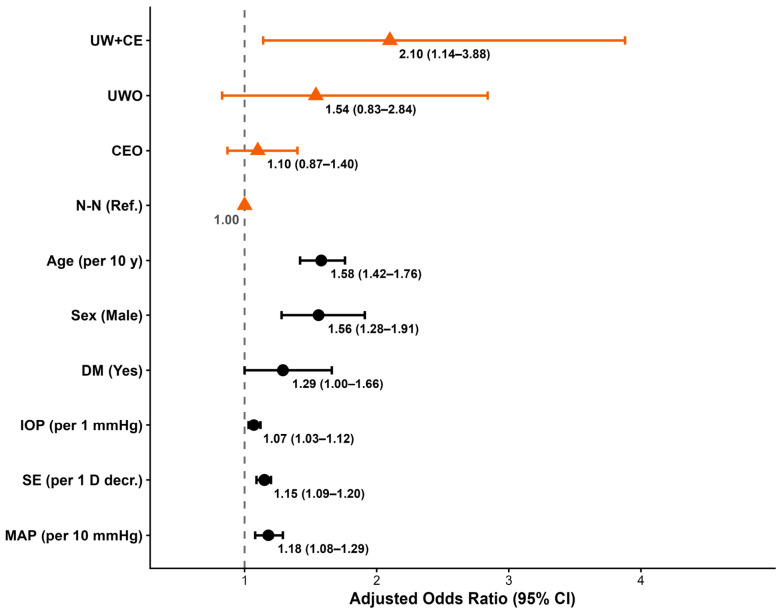
Adjusted ORs (95% CI) for OAG: multivariable logistic regression analysis (Model 2). Orange filled triangles represent the four body mass index–cold extremities groups, and black filled circles represent the covariates. The vertical dashed line indicates OR = 1.0. Abbreviations: N-N, non-underweight without cold extremities; CEO, cold extremities only; UWO, underweight only; UW+CE, underweight with cold extremities; OAG, open-angle glaucoma; OR, odds ratio; CI, confidence interval; DM, diabetes mellitus; MAP, mean arterial pressure; IOP, intraocular pressure; SE, spherical equivalent.

The results of the complex sample logistic regression analyses are summarized in Table 3 and Figure 2. In the crude analysis, the odds of OAG were significantly elevated in the UW+CE group compared to the N-N group (OR = 2.36, 95% CI: 1.26–4.43, *p* = 0.008). This association remained significant after adjusting for sex and age in Model 1 (OR = 2.07, 95% CI: 1.10–3.89, *p* = 0.024) and after further adjusting for DM, MAP, IOP, and SE in Model 2 (OR = 2.10, 95% CI: 1.14–3.88, *p* = 0.018). In contrast, neither CEO (OR = 1.10, *p* = 0.431) nor UWO (OR = 1.54, *p* = 0.170) was significantly associated with OAG compared to N-N.

The assessment of the additive interaction between cold extremities and underweight yielded the following results: RERI = 0.47 (95% CI: −1.15–2.08), AP = 0.22 (95% CI: −0.34–0.79), and S-index = 1.73 (95% CI: 0.25–11.89). Although all three measures were in the positive direction, none reached significance. The results of the multiplicative interaction test were also non-significant (*p* = 0.638) (Supplementary Table S2).

### 3.4. Association According to Alternative BMI Cutoff Values for the Definition of Underweight

**Figure 3:**
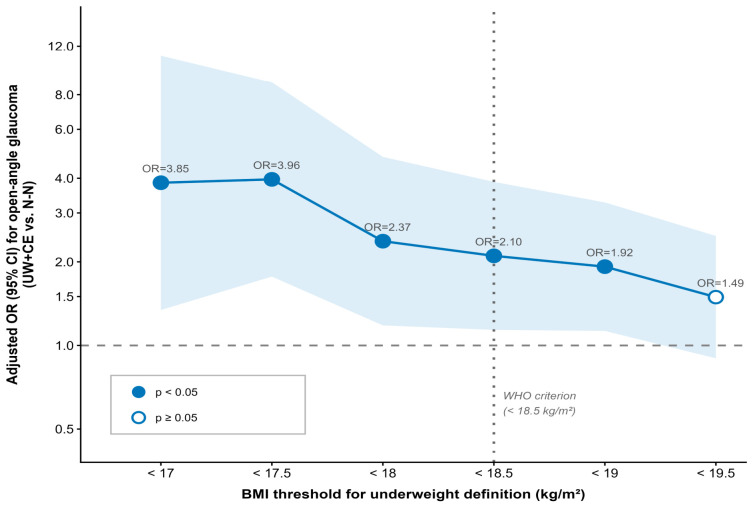
Adjusted ORs for open-angle glaucoma across varying BMI thresholds in the UW+CE group. Each data point represents the adjusted OR in the comparison between the UW+CE and N-N reference groups, which was estimated using complex sample binary logistic regression adjusted for sex, age, diabetes mellitus, mean arterial pressure, intraocular pressure, and spherical equivalent. The blue shaded band represents the 95% CI. The horizontal dashed line indicates OR = 1.0, and the vertical dotted line represents the WHO criterion for underweight (BMI < 18.5 kg/m^2^). Abbreviations: N-N, non-underweight without cold extremities; UW+CE, underweight with cold extremities; OR, odds ratio; CI, confidence interval; BMI, body mass index; WHO, World Health Organization.

When the BMI threshold for underweight was varied from <17.0 to <19.5 kg/m^2^ in increments of 0.5 kg/m^2^, the UW+CE status was consistently significantly associated with OAG from BMI < 17.0 (OR = 3.85, 95% CI: 1.34–11.07, *p* = 0.012) to BMI < 19.0 (OR = 1.92, 95% CI: 1.13–3.27, *p* = 0.016). The OR decreased progressively as the BMI threshold increased, and statistical significance was lost at BMI < 19.5 (OR = 1.49, *p* = 0.122) (Figure 3).

### 3.5. Sensitivity Analyses

In the analysis stratified by ISGEO diagnostic category, the adjusted OR for the UW+CE group was 1.78 (95% CI: 0.82–3.85, *p* = 0.143) under Category I criteria and 2.78 (95% CI: 1.16–6.68, *p* = 0.022) under Category II criteria. Both point estimates were >1; however, statistical significance was only reached for Category II (Supplementary Table S3).

In the expanded fully adjusted model that additionally incorporated hypertension, individual lipid components (hypercholesterolemia, low high-density lipoprotein cholesterolemia, and hypertriglyceridemia), migraine, alcohol consumption, smoking status, household income, education level, and physical activity, the OR in the UW+CE group remained significant at 1.93 (95% CI: 1.01–3.66, *p* = 0.046), and none of the additionally adjusted variables were significantly associated with OAG (all *p* > 0.05) (Supplementary Table S4).

## 4. Discussion

The present study provides the first population-level evidence indicating that the combination of underweight and cold extremities is independently and significantly associated with OAG, using data from the KNHANES 2008–2012. Among the 8135 participants included in the final analysis (controls: 7418; OAG: 717), those in the UW+CE group showed significantly higher odds of OAG than those in the N-N group, even after adjusting for sex, age, DM, MAP, IOP, and SE (OR = 2.10, 95% CI: 1.14–3.88, *p* = 0.018). In contrast, neither CEO (OR = 1.10, *p* = 0.431) nor UWO (OR = 1.54, *p* = 0.170) was individually significantly associated with OAG. These findings suggest that the co-occurrence of the two factors may be associated with an increased odds of OAG, a relationship that is not evident when each factor is considered independently. The novelty of this study lies in its status as the first population-level investigation of this association.

The association between underweight or low BMI and an increased risk of OAG has been consistently reported across diverse ethnic groups and study designs. Lin et al. analyzed KNHANES 2008–2011 data and reported a significantly elevated risk of OAG among individuals with BMI < 19 kg/m^2^ (OR = 2.28, 95% CI: 1.22–4.26) [19], with the association being particularly pronounced in women aged 40–49 years (OR = 8.60). In a large retrospective cohort study performed using the Korean National Health Insurance Service database (*n* = 17,000,636), Na et al. reported that underweight (BMI < 18.5 kg/m^2^) was significantly associated with a higher risk of incident POAG and identified an inverse J-shaped relationship between BMI and POAG incidence [10]. Notably, this risk was further amplified in underweight patients with concurrent diabetes (HR = 2.22), suggesting that metabolic vulnerability increases the risk of OAG conferred by being underweight. Through a multivariate analysis of KNHANES data, Kim et al. also confirmed that non-overweight status is an independent risk factor for POAG [2].

This association is not limited to the Korean population. Di Rosa et al. analyzed an African ancestry cohort from the Primary Open-Angle African American Glaucoma Genetics study (*n* = 6634) and reported that each 1 kg/m^2^ decrease in BMI was associated with a 2% increase in the risk of POAG (adjusted OR = 1.02, *p* = 0.0003). Furthermore, patients who were underweight and had POAG exhibited larger CDR values and faster visual field progression [11]. This suggests that being underweight is associated not only with POAG prevalence but also with more severe phenotypes and faster disease progression. Chen et al. conducted a retrospective cohort study using UK Biobank data (*n* = 467,768), which considered body size trajectories from early life to adulthood, and consistently found that adult low body size was associated with an increased risk of POAG (HR = 1.30, 95% CI: 1.19–1.43) [31]. Furthermore, the observation that a trajectory involving rapid weight loss from a high childhood body size to a low adulthood body size is associated with the highest risk of POAG (HR = 1.66) indicates that both the absolute level of underweight and the direction and magnitude of body weight change influence optic nerve vulnerability.

In contrast, the Tajimi Study in Japan did not identify BMI as a significant risk factor for OAG when analyzed as a continuous variable [32]. Similarly, in the present study, no significant difference in continuous BMI was observed between the glaucoma and control groups (23.87 vs. 24.16 kg/m^2^, *p* = 0.057). However, when underweight was defined as a threshold-based category (BMI < 18.5 kg/m^2^), the proportion of underweight participants was significantly larger in the glaucoma group (4.5% vs. 2.3%, *p* = 0.001), and a dose–response relationship was confirmed, with ORs increasing as the BMI threshold decreased (BMI < 19.0: OR = 1.92; BMI < 17.0: OR = 3.85). This suggests that the relationship between BMI and OAG follows a nonlinear threshold effect, whereby the risk increases only below a certain BMI cutoff, which is consistent with the inverse J-shaped relationship reported by Na et al. [10].

Several mechanisms have been proposed to explain the association between underweight and an increased risk of OAG. The most well-supported pathway involves the cerebrospinal fluid pressure (CSFP). CSFP exhibits a positive linear relationship with BMI. Fleischman et al. analyzed lumbar puncture records from 12,118 individuals and confirmed that BMI was independently and positively associated with CSFP across all age groups [33]. Low CSFP reduces the counterpressure to the IOP posterior to the optic nerve, thereby increasing the translaminar cribrosa pressure difference, which may induce anterior deformation of the lamina cribrosa and impair axonal transport, ultimately contributing to the susceptibility to optic nerve damage [34]. This mechanism is of particular clinical relevance in East Asian populations, in which NTG is the predominant subtype of POAG. A second proposed pathway involves estrogen deficiency. Adipose tissue is a major peripheral organ responsible for synthesizing estrogen from adrenal androgens, and estrogen exerts neuroprotective effects in retinal ganglion cells by activating intracellular protective signaling pathways (such as ERK-c-Fos) [35,36]. Accordingly, this protective mechanism may be attenuated in underweight individuals with insufficient adipose tissue, particularly in women in their 40s experiencing a rapid decline in estrogen around menopause, which is consistent with the markedly elevated risk in this age group reported by Lin et al. (OR = 8.60) [19]. A third pathway involves nutritional deficiency. Lee et al. analyzed KNHANES data and found that underweight Korean women with POAG (BMI < 18.5 kg/m^2^) consumed significantly lower amounts of energy, protein, fat, calcium, potassium, vitamin A, beta-carotene, thiamine, riboflavin, and vitamin C than their counterparts without POAG, suggesting that deficiencies in micronutrients with antioxidant and neuroprotective functions increase vulnerability to optic nerve damage [37]. Finally, a sarcopenia–arterial stiffness pathway may be involved. Muscle mass loss associated with being underweight may increase arterial stiffness [10], and Shim et al. reported that increased arterial stiffness was independently associated with an elevated risk of OAG (OR = 3.74) [38]. However, none of these mechanisms (reduced CSFP, estrogen deficiency, nutritional deficiency, or increased arterial stiffness) have been directly verified by simultaneously measuring the relevant biomarkers within the same population, and the relative contribution of each pathway remains to be elucidated.

Previous studies have focused on the effect of underweight or low BMI alone, and the question of how the risk of OAG may differ when underweight co-occurs with systemic vascular dysregulation has not yet been addressed. The present study differs from the existing literature in that it is the first to analyze the combined effect of underweight and cold extremities on the risk of OAG at the population level, utilizing cold extremities as a clinical marker for vascular dysregulation.

The association between cold extremities and OAG is theoretically supported by the concept of FS. FS is characterized by cold extremities as a core symptom, frequently accompanied by hypotension and low BMI, and a pathway has been proposed whereby impaired ocular blood flow autoregulation is linked to an increased risk of OAG, including NTG [16,17,39,40]. Konieczka et al. administered the FS questionnaire to 246 Korean patients with NTG and 1116 controls, and found that seven of the 15 FS symptoms were significantly more frequently reported in patients with NTG than in controls, with cold extremities being one of the significant items (*p* = 0.034) [12]. This indicates that cold extremities are significantly associated with NTG not only in Western populations but also in the Korean population, which provides the background and rationale for the present study. Additionally, in a study of patients with glaucoma (*n* = 123) classified based on four optic disc types, Broadway and Drance reported that subjective cold extremities, migraine, and peripheral vasospastic response to temperature change were all significantly more prevalent in patients with the focal ischemic disc type, and that self-reported cold extremities were strongly correlated with the vasospasm score (r = 0.82, *p* < 0.0001), demonstrating that self-reported cold extremities may serve as a clinically meaningful indicator of vascular dysregulation [41].

Nevertheless, previous studies were largely conducted using hospital-based samples, limiting their generalizability to the broader population. The present study addresses this research gap by being the first to analyze the association between cold extremities and OAG in a sample of community-dwelling Korean adults obtained through the KNHANES using a complex sampling design. The practical importance of this finding lies in the fact that cold extremities are not uncommon in the Korean adult population. In the present study, 23.3% of all participants reported cold extremities, which is consistent with previous reports of 21.6% for cold hands and 23.0% for cold feet in Korean adults [15], reconfirming that cold extremities exist at a scale warranting public health attention in the Korean population. Moreover, accumulating evidence indicates that cold extremities are not merely a subjective discomfort but serve as a clinical marker of vascular dysfunction associated with various chronic conditions. In a multicenter Korean study (*n* = 4926), Bae et al. reported that cold extremities were associated with various chronic conditions, including chronic gastritis, hypotension, and dysmenorrhea [13]. Linstra et al. analyzed the Leiden University Migraine Neuro-Analysis (LUMINA) cohort and found that cold extremities were significantly more frequently reported by female patients with migraine than by controls (OR = 2.3, 95% CI: 1.4–3.7) and that female patients with migraine with cold extremities experienced a higher frequency of attacks (4 vs. 3 episodes, *p* = 0.003), suggesting that cold extremities can function as a specific marker of vascular dysregulation vulnerability [14]. Thus, cold extremities have been reported as a clinical manifestation of vascular dysregulation across diverse organ systems, including the gastrointestinal, neurovascular, and circulatory systems. OAG, which is associated with impaired ocular blood flow regulation, can be viewed as lying within the same spectrum.

In the present study, cold extremities alone (CEO group) were not significantly associated with OAG (OR = 1.10, *p* = 0.431), whereas a significant increase in risk was observed when cold extremities co-occurred with underweight status (OR = 2.10, *p* = 0.018). This may suggest that the clinical phenotype of FS, comprising cold extremities, hypotension, and low BMI, is associated with a critical level of hemodynamic burden on ocular perfusion, primarily when its components co-occur rather than when they are present individually. Underweight may structurally reduce the mean ocular perfusion pressure [7,17] (UW+CE group: 46.56 mmHg vs. N-N group: 49.08 mmHg, *p* = 0.016), and when vascular dysregulation, represented by cold extremities, co-exists, the reduced perfusion pressure may be associated with decreased blood flow to the optic nerve head, and the resulting instability in oxygen supply has been proposed to contribute to retinal ganglion cell damage through oxidative stress and axonal transport impairment [17,39]. Conversely, in underweight patients without cold extremities (UWO group), vascular autoregulation may be relatively preserved, such that the optic nerve head blood flow is maintained relatively constant despite reduced perfusion pressure, which may explain why the risk of OAG was not significantly elevated in this group. Given that intact vascular autoregulation plays a role in maintaining relatively constant retinal and optic nerve head blood flow despite changes in perfusion pressure, the present finding that the presence or absence of cold extremities serves as a bifurcation point for the risk of OAG under the same underweight condition aligns with this physiological context [17,39,42,43]. The precise mechanisms underlying vascular dysregulation, represented by cold extremities, remain to be fully elucidated; however, autonomic nervous system function is considered to play an important role. Endothelial dysfunction has been identified as a characteristic feature of FS [17], abnormal neuro-endothelial vascular responses to cold stimulation have been reported in NTG [44], reduced autonomic activity has been associated with more severe visual field defects in OAG [45], and plasma oxytocin levels have recently been found to be reduced in patients with OAG and to correlate with the severity of visual field damage [46].

Despite this pathophysiological reasoning, a statistical interaction between cold extremities and underweight status was not confirmed in the present study. Although all three additive interaction measures were in the positive direction (RERI = 0.47, AP = 0.22, and S-index = 1.73), none reached statistical significance. The test for multiplicative interaction was also non-significant (*p* = 0.638). Therefore, based on the present findings alone, it is difficult to determine whether the elevated risk observed in the UW+CE group reflects an interaction between the two factors or an accumulation of their independent effects. This is thought to primarily reflect the limited statistical power arising from the small number of glaucoma cases in the UW+CE group (*n* = 16), and verification using larger samples is warranted in future studies. A similar pattern was observed in the sensitivity analysis stratified by the ISGEO diagnostic category, in which the UW+CE profile showed a directionally consistent association with OAG across both categories. Given the limited statistical power within each stratum, the present data do not allow a firm distinction between a genuine category-specific effect and a consistent association across both categories that reached significance only in Category II. Therefore, this stratified analysis may be more appropriately interpreted as supporting the overall robustness of the primary finding rather than as definitive evidence regarding a specific diagnostic category.

The primary findings of the present study were consistently maintained when the BMI threshold for defining underweight was varied. Across BMI cutoffs from <17.0 to <19.5 kg/m^2^ in increments of 0.5 kg/m^2^, the OR in the UW+CE group was consistently significant from BMI < 17.0 (OR = 3.85) to BMI < 19.0 (OR = 1.92) and tended to increase as the threshold was lowered. This suggests a dose–response relationship, whereby more severe underweight status confers a higher risk of OAG, which is consistent with the nonlinear threshold effect between BMI and OAG reported by Na et al. [10]. These findings support the robustness of the primary results of the present study, which are not dependent on any specific definition of underweight. Furthermore, this robustness is externally supported by the fact that previous studies consistently reported significant associations between underweight and OAG, despite applying different underweight criteria (BMI < 19 kg/m^2^ [19], BMI < 18.5 kg/m^2^ [10], and BMI as a continuous variable [11]).

This study has some limitations. First, given the cross-sectional study design, caution is required when interpreting the observed associations as causal. Second, IOP was restricted within the range of 5–21 mmHg to focus the analysis on normal-range IOP, reflecting the characteristics of East Asian populations in which NTG is the predominant POAG subtype. However, as the KNHANES IV data did not include information on previous ophthalmic diagnoses or surgical history, there is a possibility that this IOP range restriction did not completely exclude secondary glaucoma. Third, the analytical sample was restricted to participants aged ≥ 50 years. Although this restriction is considered epidemiologically and pathophysiologically justified because of the age-dependent nature of OAG and the potential mechanistic heterogeneity of cold extremities across different ages, it limits the generalizability of the present findings to younger adults. Fourth, the sequential exclusion criteria applied to ensure diagnostic clarity resulted in substantial sample attrition, which warrants caution when generalizing the findings to the broader population. Fifth, cold extremities were assessed using a self-reported questionnaire that combined cold extremities and migraine into a single item without objective verification (e.g., laser Doppler flowmetry); this combined format precluded the simultaneous inclusion of migraine as an independent covariate [47]. Sixth, central corneal thickness and gonioscopy data were unavailable in the KNHANES, and IOP and blood pressure were measured only once, precluding the assessment of diurnal variation. Seventh, a quantitative assessment of glaucoma severity was not feasible in the present study. As a large-scale population-based survey, the KNHANES employed FDT perimetry rather than standard automated perimetry, which is the gold standard for visual field assessment in clinical practice. Furthermore, the FDT results were provided as categorized ordinal data rather than continuous values, precluding the derivation of quantitative severity indices such as the mean deviation. Optical coherence tomography-based circumpapillary retinal nerve fiber layer thickness was also not incorporated into the ophthalmic examination protocol of the survey cycles used in the present analysis. Eighth, the small number of OAG cases in the UW+CE subgroup (*n* = 16) limited the statistical power to detect a significant interaction; consequently, whether the elevated odds in this group reflect a true interaction or the accumulation of independent effects could not be determined.

## 5. Conclusions

The present study provides the first population-level evidence indicating that the combination of underweight and cold extremities is independently and significantly associated with OAG prevalence in a sample representative of community-dwelling Korean adults. Analysis of data obtained from the KNHANES, which uses a complex sampling design, showed that the odds of OAG were significantly elevated in the UW+CE group compared to the N-N group, even after adjusting for sex, age, DM, MAP, IOP, and SE (OR = 2.10, 95% CI: 1.14–3.88, *p* = 0.018). This finding was consistently maintained across alternative BMI thresholds and after further adjustment for socioeconomic, lifestyle, and cardiometabolic variables. The observation that neither factor alone was significantly associated with OAG, whereas there were significantly higher odds of OAG only when both co-occurred, suggests that vascular autoregulatory dysfunction represented by cold extremities and hemodynamic vulnerability conferred by underweight status together may reflect a combined risk profile associated with optic nerve damage. These findings may serve as useful evidence for the early identification of individuals who are highly vulnerable to OAG by checking for concurrent underweight and cold extremities in clinical practice, and for the development of glaucoma screening and prevention strategies targeting this population.

## Figures and Tables

**Table 1:** Demographic and clinical characteristics according to open-angle glaucoma status.

Variables	Total	Control	Glaucoma	*p*-Value
Numbers	8135	7418	717	
Age (years)	61.15 ± 0.13	60.89 ± 0.14	63.96 ± 0.44	<0.001
Sex (%)				<0.001
Male	45.2 (0.6)	44.3 (0.6)	54.2 (2.3)	
Female	54.8 (0.6)	55.7 (0.6)	45.8 (2.3)	
Cold extremities (%)				0.685
No	76.7 (0.6)	76.8 (0.6)	76.0 (2.0)	
Yes	23.3 (0.6)	23.2 (0.6)	24.0 (2.0)	
BMI (kg/m^2^)	24.13 ± 0.04	24.16 ± 0.04	23.87 ± 0.15	0.057
BMI category (%)				0.001
≥18.5 kg/m^2^	97.5 (0.2)	97.7 (0.2)	95.5 (0.9)	
<18.5 kg/m^2^	2.5 (0.2)	2.3 (0.2)	4.5 (0.9)	
SBP (mmHg)	125.65 ± 0.29	125.24 ± 0.29	130.01 ± 0.89	<0.001
DBP (mmHg)	78.43 ± 0.17	78.33 ± 0.17	79.50 ± 0.54	<0.001
MAP (mmHg)	94.17 ± 0.19	93.97 ± 0.19	96.34 ± 0.58	<0.001
IOP (mmHg)	13.94 ± 0.05	13.89 ± 0.05	14.41 ± 0.14	<0.001
MOPP (mmHg)	48.84 ± 0.14	48.75 ± 0.14	49.81 ± 0.41	<0.001
Spherical equivalent (D)	−0.27 ± 0.02	−0.23 ± 0.02	−0.69 ± 0.14	<0.001
Myopia (%)				<0.001
No myopia	64.8 (0.7)	65.2 (0.7)	59.7 (2.2)	
Low myopia	30.0 (0.7)	30.0 (0.7)	29.9 (2.0)	
Moderate myopia	3.7 (0.3)	3.4 (0.3)	6.5 (1.3)	
High myopia	1.5 (0.2)	1.3 (0.2)	4.0 (1.0)	
Diabetes mellitus (%)				0.001
No	84.0 (0.5)	84.5 (0.5)	78.3 (2.0)	
Yes	16.0 (0.5)	15.5 (0.5)	21.7 (2.0)	

Values are presented as mean ± standard error or weighted percentage (standard error). *p*-values were calculated using the complex sample general linear model for continuous variables and the Rao-Scott chi-square test for categorical variables. Abbreviations: BMI, body mass index; SBP, systolic blood pressure; DBP, diastolic blood pressure; MAP, mean arterial pressure; IOP, intraocular pressure; MOPP, mean ocular perfusion pressure.

**Table 2:** Demographic and clinical characteristics of participants according to the underweight and cold extremities status.

Variables	N-N *n* = 5993 OAG = 535	CEO *n* = 1922 OAG = 151	*p*-Value	UWO *n* = 128 OAG = 15	*p*-Value	UW+CE *n* = 92 OAG = 16	*p*-Value
OAG (%) *							
No	91.7 (0.5)	91.6 (0.8)		86.0 (3.8)		82.3 (4.6)	
Yes	8.3 (0.5)	8.4 (0.8)		14.0 (3.8)		17.7 (4.6)	
Age (years) ***	60.90 ± 0.15	61.46 ± 0.24	0.039	66.47 ± 1.06	<0.001	65.44 ± 1.33	0.001
Sex (%) ***							
Male	49.5 (0.7)	29.2 (1.3)	<0.001	71.3 (4.9)	<0.001	36.4 (5.9)	0.037
Female	50.5 (0.7)	70.8 (1.3)		28.7 (4.9)		63.6 (5.9)	
BMI (kg/m^2^) ***	24.48 ± 0.05	23.68 ± 0.08	<0.001	17.60 ± 0.09	<0.001	17.48 ± 0.12	<0.001
SBP (mmHg) *	126.02 ± 0.32	124.70 ± 0.53	0.059	121.68 ± 2.41	0.148	124.39 ± 3.11	0.596
DBP (mmHg) ***	78.87 ± 0.19	77.38 ± 0.30	<0.001	73.99 ± 1.14	<0.001	74.94 ± 1.11	<0.001
MAP (mmHg) ***	94.58 ± 0.21	93.15 ± 0.34	<0.001	89.88 ± 1.46	0.003	91.42 ± 1.50	0.035
IOP (mmHg) *	13.98 ± 0.06	13.82 ± 0.08	0.131	13.32 ± 0.30	0.077	14.39 ± 0.33	0.221
MOPP (mmHg) ***	49.08 ± 0.15	48.28 ± 0.24	0.004	46.60 ± 1.02	0.016	46.56 ± 0.96	0.016
Spherical equivalent (D)	−0.28 ± 0.03	−0.22 ± 0.05	0.836	−0.17 ± 0.18	1.000	−0.27 ± 0.51	1.000
Myopia (%)							
No myopia	64.3 (0.8)	66.3 (1.3)		63.4 (5.5)		70.9 (5.8)	
Low myopia	30.3 (0.8)	29.5 (1.3)		30.2 (5.1)		22.9 (5.2)	
Moderate myopia	4.0 (0.3)	2.6 (0.5)		5.9 (3.3)		2.7 (1.8)	
High myopia	1.5 (0.2)	1.7 (0.4)		0.5 (0.5)		3.5 (3.3)	
Diabetes mellitus (%)							
No	83.4 (0.6)	85.5 (0.9)		89.8 (3.0)		89.6 (4.3)	
Yes	16.6 (0.6)	14.5 (0.9)		10.2 (3.0)		10.4 (4.3)	

Values are presented as mean ± standard error or weighted percentage (standard error). Asterisks indicate overall group differences (* *p* < 0.05, *** *p* < 0.001) assessed using the complex sample general linear model or the Rao-Scott chi-square test. Pairwise *p*-values vs. N-N are shown for continuous variables with sequential Bonferroni correction; for categorical variables, only the overall significance is indicated. The overall *p*-value for OAG prevalence was 0.016. Abbreviations: N-N, non-underweight without cold extremities; CEO, cold extremities only; UWO, underweight only; UW+CE, underweight with cold extremities; OAG, open-angle glaucoma; BMI, body mass index; SBP, systolic blood pressure; DBP, diastolic blood pressure; MAP, mean arterial pressure; IOP, intraocular pressure; MOPP, mean ocular perfusion pressure.

**Table 3:** ORs (95% CI) for open-angle glaucoma according to underweight and cold extremities status.

Groups	Crude		Model 1		Model 2	
	OR (95% CI)	*p*-Value	OR (95% CI)	*p*-Value	OR (95% CI)	*p*-Value
N-N	Ref.		Ref.		Ref.	
CEO	1.00 (0.80–1.26)	0.974	1.07 (0.84–1.36)	0.574	1.10 (0.87–1.40)	0.431
UWO	1.80 (0.98–3.30)	0.060	1.30 (0.71–2.39)	0.387	1.54 (0.83–2.84)	0.170
UW+CE	**2.36 (1.26–4.43)**	**0.008**	**2.07 (1.10–3.89)**	**0.024**	**2.10 (1.14–3.88)**	**0.018**

ORs and *p*-values were estimated using complex sample binary logistic regression. Model 1: adjusted for sex and age. Model 2: additionally adjusted for diabetes mellitus, mean arterial pressure, intraocular pressure, and spherical equivalent. Bold values indicate statistical significance (*p* < 0.05). Abbreviations: N-N, non-underweight without cold extremities; CEO, cold extremities only; UWO, underweight only; UW+CE, underweight with cold extremities; OR, odds ratio; CI, confidence interval.

## Data Availability

The data analyzed in this study are publicly available from the Korea National Health and Nutrition Examination Survey (KNHANES), administered by the Korea Disease Control and Prevention Agency (KDCA). The raw data can be freely accessed upon researcher application at https://knhanes.kdca.go.kr/ (accessed on 4 June 2026).

## Supplementary Materials

The following supporting information can be downloaded at: https://www.mdpi.com/article/10.3390/medicina62071411/s1, Supplementary Table S1: Comparison of general and clinical characteristics between the excluded and included participants among the KNHANES 2008–2012 participants aged ≥ 50 years; Supplementary Table S2: Assessment of additive interaction between cold extremities and underweight status on the risk of open-angle glaucoma; Supplementary Table S3: Adjusted odds ratios for open-angle glaucoma stratified by ISGEO diagnostic category (Category I and Category II); Supplementary Table S4: Adjusted ORs for open-angle glaucoma: comparison between the primary model (Model 2) and the fully adjusted model, including socioeconomic, lifestyle, and cardiometabolic variables.

## Abbreviations

The following abbreviations are used in this manuscript:

POAG	primary open-angle glaucoma
OAG	open-angle glaucoma
IOP	intraocular pressure
NTG	normal-tension glaucoma
BMI	body mass index
KNHANES	Korea National Health and Nutrition Examination Survey
KDCA	Korea Disease Control and Prevention Agency
IRB	Institutional Review Board
SE	spherical equivalent
CDR	cup-to-disc ratio
RNFL	retinal nerve fiber layer
ISNT	inferior-superior-nasal-temporal
VF	visual field
FDT	frequency doubling technology
SBP	systolic blood pressure
DBP	diastolic blood pressure
MAP	mean arterial pressure
MOPP	mean ocular perfusion pressure
N-N	non-underweight without cold extremities
CEO	cold extremities only
UWO	underweight only
UW+CE	underweight with cold extremities
RERI	relative excess risk due to interaction
AP	attributable proportion due to interaction
S-index	synergy index
CI	confidence interval
OR	odds ratio
VIF	variance inflation factor
CSFP	cerebrospinal fluid pressure
