# Supplementary material for: Cold Extremities and Underweight as a Combined Risk Profile for Open-Angle Glaucoma: A Population-Based Cross-Sectional Study in Korea
Source: Medicina (Kaunas). 2026 Jul 21;62(7):1411. doi: 10.3390/medicina62071411 (PMC13414082; doi:10.3390/medicina62071411)
Supplement: Supplementary file 1 [file medicina-62-01411-s001.zip › medicina-4388552-supplementary.pdf]

Supplementary Table S1. Comparison of general and clinical characteristics between the excluded and included participants among the KNHANES 2008–2012 participants aged ≥50 years.

Variables	Excluded (n = 8,982)	Included (n = 8,135)	p-value
Age (years)	63.59 ± 0.21	61.15 ± 0.14	**< 0.001**
BMI (kg/m²)	23.87 ± 0.05	24.13 ± 0.04	**< 0.001**
SBP (mmHg)	127.44 ± 0.33	125.65 ± 0.29	**< 0.001**
DBP (mmHg)	77.79 ± 0.19	78.43 ± 0.17	**0.003**
MAP (mmHg)	94.34 ± 0.21	94.17 ± 0.19	0.468
IOP (mmHg)	14.04 ± 0.07	13.94 ± 0.05	0.105
MOPP (mmHg)	48.88 ± 0.15	48.84 ± 0.14	0.816
Spherical equivalent (D)	−0.229 ± 0.029	−0.268 ± 0.024	0.271
Sex (%)			**0.009**
Male	47.7 (0.7)	45.2 (0.6)	
Female	52.3 (0.7)	54.8 (0.6)	
Diabetes mellitus (%)			**0.007**
Yes	18.2 (0.7)	16.0 (0.5)	
No	81.8 (0.7)	84.0 (0.5)	
Myopia grade (%)			0.405
Normal/hyperopia	65.8 (0.8)	64.8 (0.7)	
Low myopia	28.6 (0.7)	30.0 (0.7)	
Moderate myopia	4.0 (0.3)	3.7 (0.3)	
High myopia	1.7 (0.2)	1.5 (0.2)	
BMI category (%)			**0.006**
Non-underweight (≥18.5)	96.6 (0.3)	97.5 (0.2)	
Underweight (<18.5)	3.4 (0.3)	2.5 (0.2)	
Cold extremities (%)			0.662
Yes	23.7 (0.7)	23.3 (0.6)	
No	76.3 (0.7)	76.7 (0.6)	
BMI-cold extremities groups (%)			0.060
N-N (Reference)	74.3 (0.7)	75.3 (0.6)	
CEO	22.3 (0.7)	22.2 (0.6)	
UWO	2.0 (0.2)	1.4 (0.2)	
UW+CE	1.3 (0.2)	1.1 (0.1)	

Among the KNHANES 2008–2012 participants aged ≥50 years (n = 17,117), those excluded due to missing data in any analytical variable, ineligible anterior chamber angle or IOP findings, or unresolved glaucoma suspect status were assigned to the 'Excluded' group (n = 8,982), whereas participants included in the final analytical sample comprised the 'Included' group (n = 8,135). Values are presented as weighted mean ± standard error or weighted percentage (standard error). p-values were calculated using the complex sample general linear model for continuous variables and the Rao-Scott chi-square test for categorical variables. Bold p-values indicate statistical significance ($p < 0.05$). Abbreviations: BMI, body mass index; SBP, systolic blood pressure; DBP, diastolic blood pressure; MAP, mean arterial pressure; IOP, intraocular pressure; MOPP, mean ocular perfusion pressure; N-N, non-underweight without cold extremities; CEO, cold extremities only; UWO, underweight only; UW+CE, underweight with cold extremities.

Supplementary Table S2. Assessment of additive interaction between cold extremities and underweight status on the risk of open-angle glaucoma.

Measure	Estimate	95% CI
RERI	0.47	−1.15 to 2.08
AP	0.22	−0.34 to 0.79
S-index	1.73	0.25 to 11.89

Additive interaction between cold extremities and underweight status on the risk of open-angle glaucoma was assessed using the RERI, AP, and S-index. All measures were derived from the adjusted odds ratios of the four BMI-cold extremities groups (Model 2), using the delta method for 95% CI estimation. RERI > 0, AP > 0, and S-index > 1 indicate positive additive interaction. None of the measures reached statistical significance (all 95% CIs include the null value). Abbreviations: RERI, relative excess risk due to interaction; AP, attributable proportion due to interaction; S, synergy index; CI, confidence interval; UW+CE, underweight with cold extremities.

Supplementary Table S3. Adjusted odds ratios for open-angle glaucoma stratified by ISGEO diagnostic category (Category I and Category II).

Group	Category I Glaucoma		Category II Glaucoma	
	**OR (95% CI)**	**P Value**	**OR (95% CI)**	**P Value**
No. of glaucoma cases	427		290	
N-N (Reference)	Ref.		Ref.	
CEO	1.10 (0.80–1.52)	0.550	1.12 (0.81–1.54)	0.510
UWO	1.71 (0.79–3.70)	0.175	1.23 (0.49–3.06)	0.658
UW+CE	1.78 (0.82–3.85)	0.143	**2.78 (1.16–6.68)**	**0.022**

Category I: structural and functional evidence of glaucoma; Category II: structural evidence only, based on ISGEO criteria. ORs and 95% CIs were estimated using complex sample logistic regression, adjusted for sex, age, diabetes mellitus, mean arterial pressure, intraocular pressure, and spherical equivalent. Bold values indicate statistical significance ($p < 0.05$). Abbreviations: N-N, non-underweight without cold extremities; CEO, cold extremities only; UWO, underweight only; UW+CE, underweight with cold extremities; OR, odds ratio; CI, confidence interval; ISGEO, International Society of Geographical and Epidemiological Ophthalmology.

Supplementary Table S4. Adjusted ORs for open-angle glaucoma: comparison between the primary model (Model 2) and the fully adjusted model, including socioeconomic, lifestyle, and cardiometabolic variables.

Variables	Model 2[a] OR (95% CI)	p-Value	Full Model[b] OR (95% CI)	p-Value
Analytic sample, No.	8,135		7,957	
**BMI-cold extremities groups**				
N-N (Reference)	Ref.		Ref.	
CEO	1.10 (0.87–1.40)	0.431	1.07 (0.84–1.38)	0.571
UWO	1.54 (0.83–2.84)	0.170	1.63 (0.86–3.09)	0.135
UW+CE	**2.10 (1.14–3.88)**	**0.018**	**1.93 (1.01–3.66)**	**0.046**
**Additional covariates (Full Model only)**				
Alcohol consumption (yes vs no)			1.02 (0.81–1.30)	0.852
Current smoking (yes vs no)			0.89 (0.66–1.20)	0.455
Household income				
Low vs High			1.22 (0.88–1.69)	0.242
Middle-low vs High			0.96 (0.70–1.31)	0.779
Middle-high vs High			0.94 (0.68–1.30)	0.721
Education level				
Elementary or less vs College+			0.98 (0.69–1.40)	0.914
Middle school vs College+			1.04 (0.71–1.52)	0.826
High school vs College+			1.17 (0.81–1.69)	0.390
Physical activity (MET, per unit)			1.00 (1.00–1.00)	0.542
Hypercholesterolemia (yes vs no)[c]			1.11 (0.88–1.41)	0.383
Low HDL cholesterolemia (yes vs no)[d]			1.01 (0.79–1.29)	0.950
Hypertriglyceridemia (yes vs no)[e]			1.15 (0.88–1.49)	0.299
Migraine (yes vs no)[f]			0.94 (0.74–1.21)	0.649
Hypertension (yes vs no)[g]			0.97 (0.77–1.23)	0.813

[a] Model 2: adjusted for sex, age, diabetes mellitus, mean arterial pressure, intraocular pressure, and spherical equivalent (n = 8,135).

[b] Full Model: additionally adjusted for alcohol consumption, current smoking, household income, education level, physical activity (MET), hypercholesterolemia, hypertension, low HDL cholesterolemia, hypertriglyceridemia, and migraine (n = 7,957 owing to missing data for additional variables).

[c] Hypercholesterolemia was defined as a fasting total cholesterol level ⩾ 240 mg/dL or current use of cholesterol-lowering medication.
[d] Low HDL cholesterolemia was defined as HDL cholesterol < 40 mg/dL.
[e] Hypertriglyceridemia was defined as triglycerides ≥ 200 mg/dL.
[f] Migraine was evaluated using the same self-reported questionnaire item as for cold extremities.
[g] Hypertension was defined as a systolic blood pressure ≥ 140 mmHg or diastolic blood pressure ≥ 90 mmHg, or current use of antihypertensive medication.
The full model was not used as the primary analysis because the increased number of covariates relative to the limited number of glaucoma events in the UW+CE group (n = 16) may violate the events-per-variable principle and increase the risk of model overfitting. None of the additional variables showed statistically significant associations with open-angle glaucoma in the full model (all $p > 0.05$). Abbreviations: N-N, non-underweight without cold extremities; CEO, cold extremities only; UWO, underweight only; UW+CE, underweight with cold extremities; OR, odds ratio; CI, confidence interval; MET, metabolic equivalent of task; HDL, high-density lipoprotein. Bold values indicate statistical significance ($p < 0.05$).
